# Effect of magnesium on vascular calcification in chronic kidney disease patients: a systematic review and meta-analysis

**DOI:** 10.1080/0886022X.2023.2182603

**Published:** 2023-03-01

**Authors:** Ya Zhan, Rongjia Zhang, Guisen Li

**Affiliations:** a Renal Department, The Third Hospital of Mianyang, Sichuan Mental Health Center, Mianyang, China; b Department of Clinical Medicine, North Sichuan Medical College, Nanchong, China; c Renal Department, Sichuan Provincial People’s Hospital, School of Medicine, University of Electronic Science and Technology of China, Chengdu, China

**Keywords:** Magnesium, vascular calcification, chronic kidney disease, meta-analysis

## Abstract

**Purpose:**

To evaluate the effects of magnesium (Mg) supplementation on vascular calcification (VC) in patients with chronic kidney disease (CKD).

**Methods:**

PubMed, Embase, Cochrane Library, Medline, Web of Science, CNKI, VIP, and WanFang databases were searched from build to July 2022. Randomized controlled trials (RCT) and non-RCT related to whether Mg supplementation inhibits VC in patients with CKD were included. The literature was screened according to inclusion and exclusion criteria, and quality evaluation and data collection were performed. Meta-analysis was performed using Review Manager 5.4 software.

**Results:**

8 RCTs and 1 non-RCT studies with a total of 496 patients were eventually included. Compared to control groups, Mg supplementation increased serum Mg levels (SMD = 1.26, 95% CI: −0.70 to 1.82, *p* < 0.001), but it was not statistically significant in alleviating the degree of VC, increasing T50, and reducing serum phosphorus (P) levels in patients with CKD (all *p* > 0.05). Oral Mg reduced left (WMD=−0.06, 95% CI. −0.11 to −0.01, *p* = 0.03) and right (WMD=−0.07, 95% CI: −0.13 to −0.01, *p* = 0.02) carotid intima-media thickness (cIMT). Additionally, calcium (Ca) (SMD=−0.43, 95% CI: −0.74 to −0.11, *p* = 0.008) and parathyroid hormone (PTH) (SMD=−0.43, 95% CI: −0.75 to −0.11, *p* = 0.008) levels were reduced by increasing dialysate Mg concentration.

**Conclusions:**

Mg supplementation increased serum Mg levels and reduced Ca, PTH, and cIMT, but it did not reduce VC scores in patients with CKD. This still requires further studies with larger samples to evaluate the effect of Mg supplementation on VC.

## Introduction

Chronic kidney disease (CKD) is a common and public disease that endangers human health worldwide. As kidney disease progresses, a variety of complications can occur, and cardiovascular disease is one of the most common and serious complications, especially vascular calcification (VC). A meta-analysis including 9883 patients with the end-stage renal disease showed that the overall prevalence of abdominal aortic calcification in dialysis patients was 68.5% in different regions worldwide [1], and the China Dialysis Calcification Study showed that the prevalence of VC in Chinese CKD dialysis patients was 77.4% [2]. VC significantly increases the risk of all-cause mortality and cardiovascular death in patients with CKD compared to patients without VC [3,4]. Intervening in the progression of VC is important to improve the quality of life and prolong the survival of patients, but there is a lack of effective medications for the treatment of VC in patients with CKD, and the main focus is on removing the causative factors and risk factors [5].

Mg is an important cation in the human body and is essential in many biological processes, improving vascular endothelial function, inhibiting inflammation and oxidative stress, and anti-atherosclerosis [6]. There are large individual differences in serum Mg levels in patients with CKD due to reduced renal function. Several studies have shown that low serum Mg levels increase mortality in patients with CKD and that high serum Mg is beneficial for survival in hemodialysis (HD) patients [7,8]. There is a correlation between serum Mg and VC, metabolic disorder of calcium (Ca) and phosphorus (P), and secondary hyperparathyroidism. Mg is involved in VC through a variety of molecular mechanisms. On the one hand, Mg^2+^ replaces Ca ions in the structure of hydroxyapatite [9], leading to a loss of its crystallinity and inhibiting the maturation of calmodulin particles, thus inhibiting VC [10]. On the other hand, Mg inhibits the wnt/β-catenin signaling pathway to reduce VC [11]. In animal studies, a high-Mg diet prevented aortic calcification in 5/6 nephrectomized mice [12] as well as in Klotho knockout mice [13]. Several studies are launched to demonstrate whether Mg supplementation can inhibit the progression of VC in patients with CKD. Sakaguchi et al. [14] showed that Mg supplementation may inhibit VC to some extent. However, the findings of Srisuwarn et al. [15] and Milicevic et al. [16] showed that Mg supplementation did not significantly alleviate the progression of VC in HD patients. The findings among the above studies are controversial and are mostly small sample studies. Therefore, this study mainly collected relevant research data and used meta-analysis to investigate the effect of Mg supplementation on VC and related markers in patients with CKD.

## Materials and methods

### Data sources, search strategy, and selection criteria

This Meta-analysis was reported in accordance with The Priority Reporting Entries for Systematic Evaluation and Meta-Analysis: a PRISMA Statement [17] and has been registered in the Prospero system (CRD42022351986).

Computer searches were conducted on Pubmed, Embase, Cochrane, Web of Science, Medline, CNKI, VIP, CBM, and WanFang databases, respectively, for the period from the creation of each database to July 2022. A combination of Mesh terms and free words were used for the search. Search terms include chronic kidney disease, kidney disease, chronic renal insufficiency, renal insufficiency, chronic renal failure, kidney failure, renal failure, chronic kidney insufficiency, kidney insufficiency, chronic nephropathy, chronic renal disease, chronic kidney failure, end stage renal disease, end stage kidney disease, renal replacement therapy, predialysis, dialysis, renal dialysis, uremia, hemodialysis, hemodialysis, hemofiltration, haemofiltration, hemodiafiltration, haemodiafiltration, peritoneal dialysis, CKD, CRF, CRD, CKF, ESKD, ESRD, ESKF, ESRF, HD, PD, end stage renal failure, end stage kidney failure, vascular calcification, blood vessel calcification, arterial calcification, aorta calcification, coronary artery calcification, valve calcification, calcified blood vessel, calcified vasculature, calcified artery, alcified valve, vascular calcinosis, vascular calcinoses, abdominal aortic calcification, cardiac valvular calcification, valvular calcification and magnesium. Simultaneous manual search of references for relevant reviews or systematic evaluations.

Inclusion Criteria: (1) Study population was aged ≥18 years with CKD, including HD, peritoneal dialysis, and renal transplantation patients. (2) The patients in the treatment group were treated with Mg (It can be in any form of Mg supplementation including medications, dialysis prescriptions, and diets). The patients in the control group were given a placebo or blank control. Studies with combined interventions in both groups were allowed, but other measures remained consistent between the two groups. (3) Study type is RCT or non-RCT. (4) Data are complete and detailed information is provided before and after treatment, including coronary artery calcification (CAC) scores, Ca, P, parathyroid hormone (PTH), serum calcification tendency (T50), and carotid intima-media thickness (cIMT).

Exclusion Criteria: (1) The type of literature is retrospective studies, case reports, systematic evaluations and meta-analyses, reviews, cross-sectional and case-control studies. (2) Study populations were patients with acute kidney injury or non-CKD. (3) The detailed data is incomplete or cannot be extracted. (4) Repeatedly published studies.

### Data collection and quality assessment

Two reviewers (Yz and Rjz, respectively) independently screened the literature and extracted data. Disagreements or uncertainties between two reviewers were discussed and agreed upon, or a third investigator was involved in the decision. If the original literature data could not be extracted, the first author of the article was contacted and the literature was excluded if contact could not be made. The data were collected as follows: (1) General information: first author, year of publication, country; (2) Basic study characteristics: sample size, patient’s age, vintage year, intervention and control group measures, and follow-up time; (3) Evaluation indicators: baseline value, endpoint value, and change value. The data of kappa is 0.856 during the systematic searches.

Two reviewers evaluated the quality of the included RCTs and non-RCT studies. RCTs were evaluated for quality according to the modified Jadad scale [18]. Scoring included random sequence generation, randomization hiding, blinding, and description of withdrawal. A score of 0–3 was considered low quality and a score of 4–7 was considered high quality. Non-RCT studies were evaluated for quality according to the Minors scale [19].

### Statistical analysis

Review Manager 5.4 was used for analysis. The weighted or standard mean difference (WMD or SMD) and 95% confidence interval (CI) were used as effect sizes for the measurement information. Subgroup analysis was performed based on the mode of administration (dialysis prescriptions or oral medications). Heterogeneity among studies was tested by using I2. If I2 ≥ 50% or p ≤ 0.1, heterogeneity among studies was considered to exist and a random-effects model was used for analysis, otherwise a fixed-effects model was used. *p* < 0.05 was deemed to be statistically significant.

## Results

### Literature search

**Figure 1.**
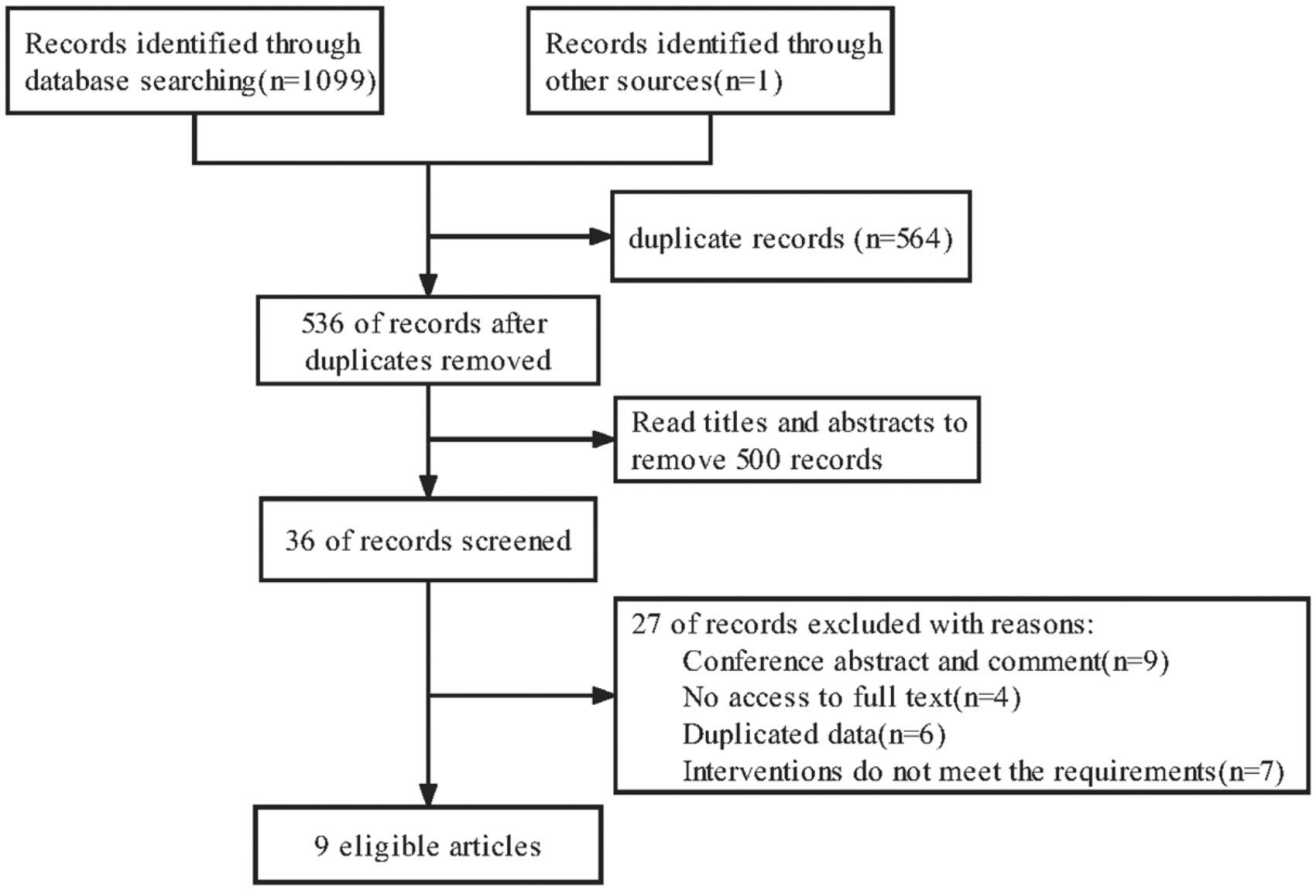
Flow diagram of study selection.

A total of 9 studies (8 RCTs and 1 non-RCT studies) were included. The specific literature screening process is shown in Figure 1. The main characteristics of the nine studies are shown in Table 1. A total of 496 patients were included, of which 157 patients were intervened by increasing the concentration of Mg prescribed for dialysis.

**Table 1.** Baseline characteristic of included studies.

Author, year	Country	Population	Sample size	Duration of HD		Intervention	Control	FU	Outcomes	Design	Jade/ Minors
				Intervention	Control						
Turgut et al. 2008[34]	Turkey	HD	44	41.3 ± 28.8^a^ (months)	19.5 ± 9.2 (months)	610 mg Mg citrate, every other day	Ca acetate	2 months	cIMT, Ca, P, iPTH	RCT	3
Mortazavi et al. 2013[35]	Iran	HD	52	4.53 ± 3.06^a^ (years)	4.52 ± 2.55 (years)	440 mg Mg oxide, three times a week	Placebo	6 months	cIMT, Ca, P, iPTH	RCT	6
Tzanakis et al. 2014[24]	Greece	HD	59	40 ± 49^a^ (months)	37 ± 56 (months)	OsvaRen (435 mg Ca acetate combined with 235 mg Mg carbonate per tablet), three tablets daily	600 mg Ca acetate, three tablets daily	12 months	Number of patients with improved VCS, Ca, P, iPTH	RCT	5
Bressendorff et al. 2016[36]	Denmark	CKD 3–4 stage	34	/	/	Slow-release Mg hydroxide 360 mg once daily and twice daily	Placebo once daily	8 weeks	T50, ionCa, P, iPTH	RCT	5
Bressendorff et al. 2018[26]	Denmark	HD	57	39 (21–99)^b^ (months)	30 (13–59) (months)	High dialysate Mg (2.0 mEq/L)	Standard dialysate Mg (1.0 mEq/L)	42 days	T50, ionCa, P, iPTH	RCT	7
Sakaguchi et al. 2019 [14]	Japan	CKD 3–4 stage	96	/	/	330mg Mg oxide per day	Standard therapy for CKD	2 years	CACS, Ca, P, iPTH	RCT	4
Talari et al. 2019 [37]	Iran	HD	54	4.0 ± 1.0^b^ (years)	3.8 ± 1.0 (years)	250 mg/d Mg supplements as Mg oxide	Placebo	24 weeks	cIMT	RCT	5
Srisuwarn et al. 2022 [15]	Thailand	HD	40	80（44–105)^b^ (months)	83（38-128） (months)	High dialysate Mg (1.75 mEq/L)	Standard dialysate Mg (0.7 mEq/L)	26 weeks	CACS, Ca, P, iPTH	Non-RCT	21
Milicevic et al. 2022 [16]	Serbia	HD	60	28.97 ± 14.63^b^ (months)	23.00 ± 14.66 (months)	Dialysate-Mg level (1 mmol/L)	Dialysate-Mg level (0.5 mmol/L)	12 months	CACS, Ca, P, PTH	RCT	2

FU: follow-up; HD: hemodialysis; CKD: chronic kidney disease; Mg: magnesium; Ca: calcium; P: phosphorus; iPTH: intact parathyroid hormone; ionCa: ionized calcium; cIMT: carotid intima-media thickness; CACS: coronary artery calcium score; VCS: vascular calcification score; T50: serum calcification propensity; RCT: randomized controlled study.

^a^Mean ± standard deviation; ^b^Medians and interquartile ranges.

### Effect of Mg on serum Mg level

**Figure 2.**
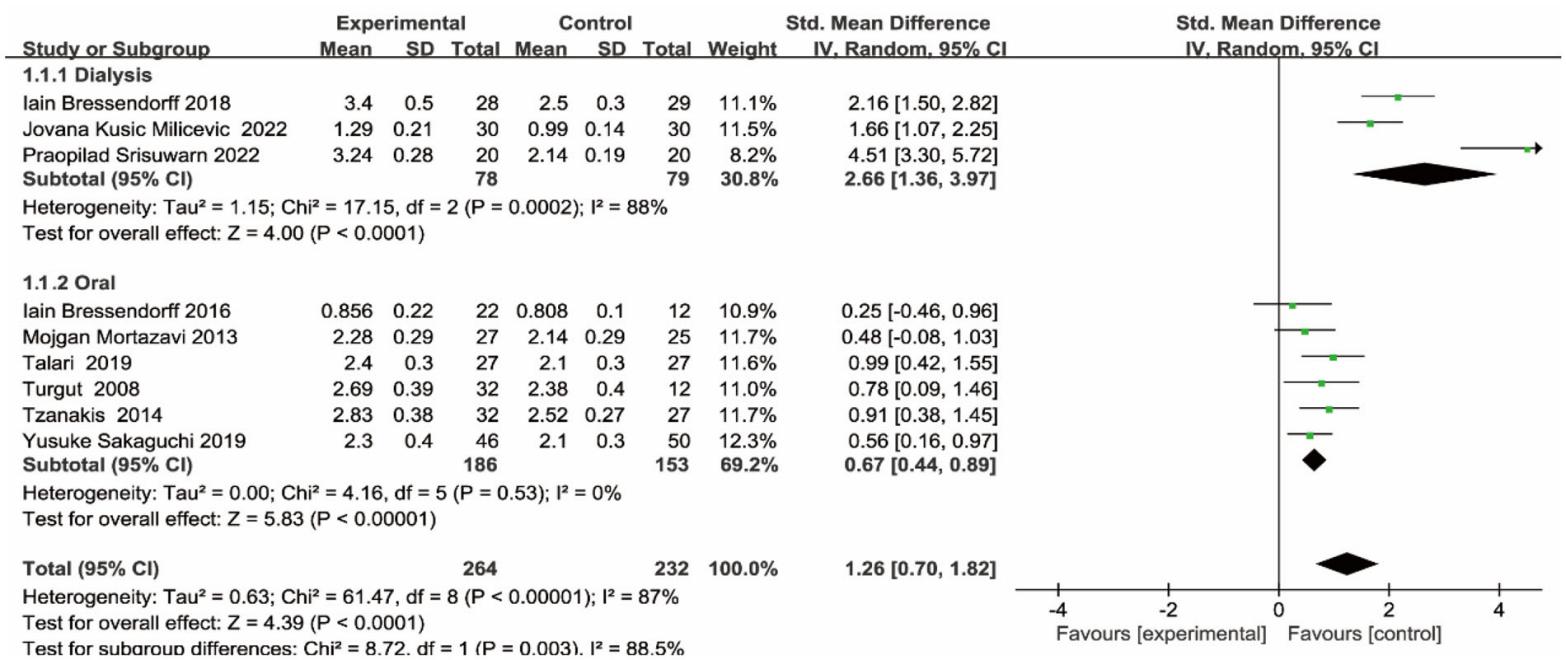
Effect of magnesium supplementation on serum magnesium level.

As shown in Figure 2, all studies provided changes in serum Mg levels before and after treatment, with statistical heterogeneity between studies (I2 = 87%, *p* < 0.001), and were analyzed using a random effects model. The results showed that serum Mg levels increased after Mg supplementation compared to the control group (SMD = 1.26, 95% CI: 0.70 to 1.82, *p* < 0.001). A subgroup analysis was done depending on the intervention type (oral and dialysis). Both oral administration (SMD = 0.67, 95% CI: 0.44 to 0.89, *p* < 0.001) and increase of Mg concentrations in dialysate (SMD = 2.66, 95% CI: 1.36 to 3.97, *p* < 0.001) could increase the serum Mg levels of CKD patients.

### Effect of Mg on VC

**Figure 3.**
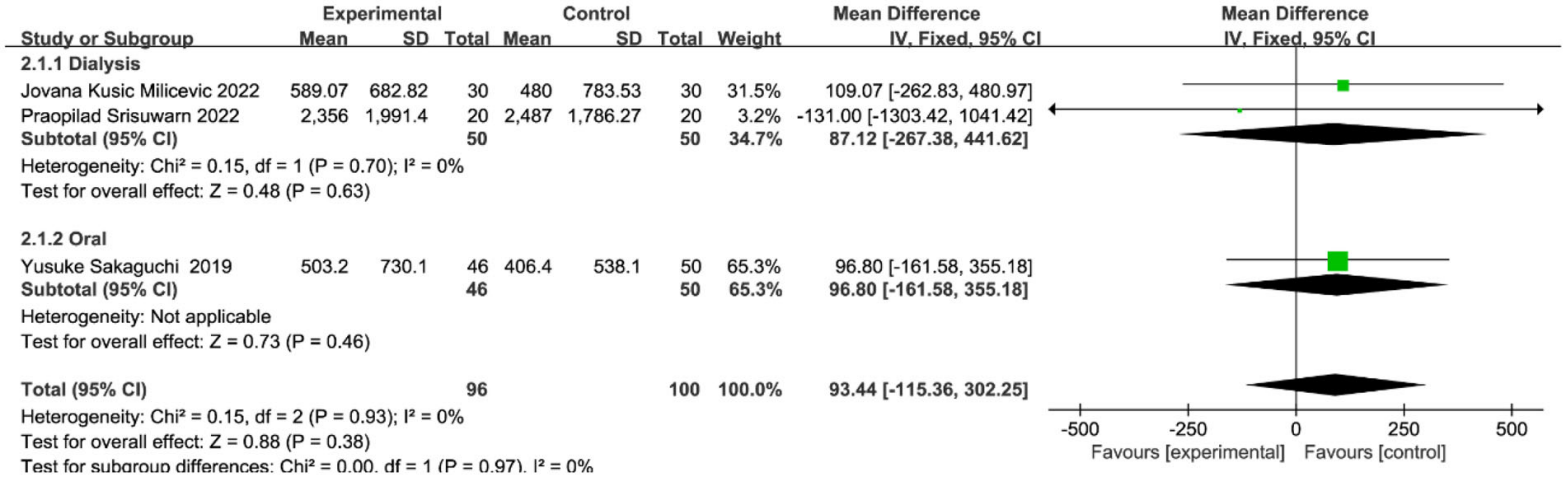
Effect of magnesium supplementation on vascular calcification scores.

Three studies provided quantitative measures of VC scores before and after treatment. All three studies used computed tomography to determine CAC scores. A total of 196 patients were included, with 96 and 100 patients in treatment and control groups, respectively. There was no statistical heterogeneity between studies (I2 = 0%, *p* = 0.93) and a fixed-effects model was used for analysis. The results showed that there was no statistical difference between the two groups (WMD = 93.44, 95% CI: -115.36 to 302.25, *p* = 0.38) (Figure 3), indicating that the change in CAC scores in patients after Mg supplementation was not significant. Subsequent subgroup analysis showed that there were no significant changes in CAC scores whether patients receiving oral administration or dialysate magnesium supplements.

**Figure 4.**
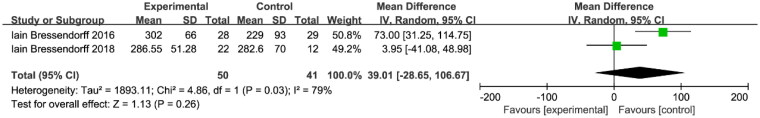
Effect of magnesium supplementation on T50. T50, serum calcification propensity.

Two studies reported the effect of Mg supplementation on T50. There were 91 patients in total, including 50 patients in the treatment group. There was statistical heterogeneity between the two studies (I2 = 79%, *p* = 0.03) and no statistical difference between the two groups when analyzed by using a random effects model (SMD = 39.01, 95% CI: -28.65 to 106.67, *p* = 0.26) (Figure 4).

### Effect of Mg on Ca, P, and PTH levels in patients with CKD

**Figure 5.**
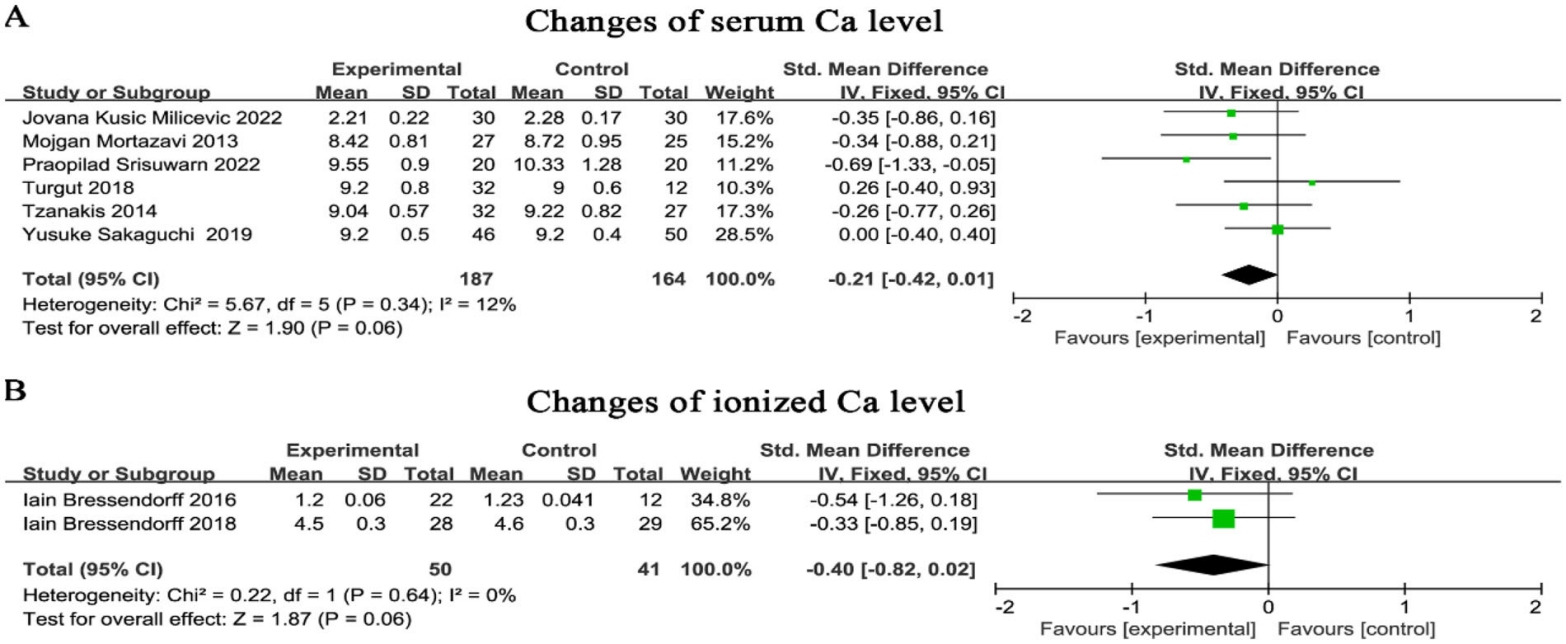
Effect of magnesium supplementation on serum Ca (A) and ionized Ca (B) levels. Ca, calcium.

A total of eight studies provided pre- and post-treatment assay values for Ca, six of which were serum Ca and two of which were ionized Ca. The results showed no significant effect of Mg supplementation on serum Ca (SMD=−0.21, 95% CI: −0.42 to 0.01, *p* = 0.06) (Figure 5(A)) and ionized Ca (SMD=−0.40, 95% CI: −0.82 to 0.02, *p* = 0.06) (Figure 5(B)).

**Figure 6.**
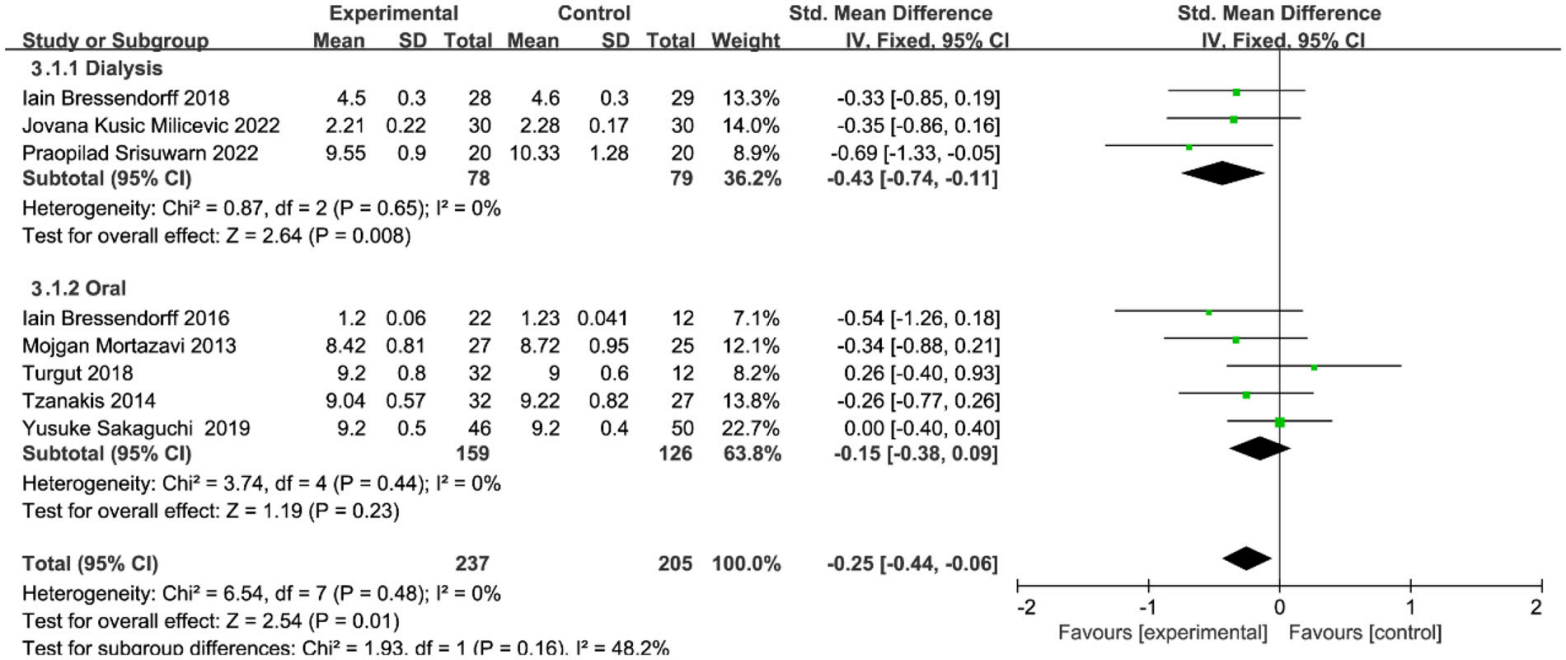
Subgroup analysis of magnesium supplementation on Ca level. Ca, calcium.

Subgroup analysis of Ca by means of Mg supplementation (oral or dialysis) was performed and there was no heterogeneity between studies. Mg supplementation via oral medication had no significant effect on Ca (SMD=−0.15, 95% CI: −0.38 to 0.09, *p* = 0.23) (Figure 6). However, Ca concentration was reduced by increasing Mg concentration in the dialysis prescription compared to the control group (SMD=−0.43, 95% CI: −0.74 to −0.11, p = 0.008) (Figure 6).

**Figure 7.**
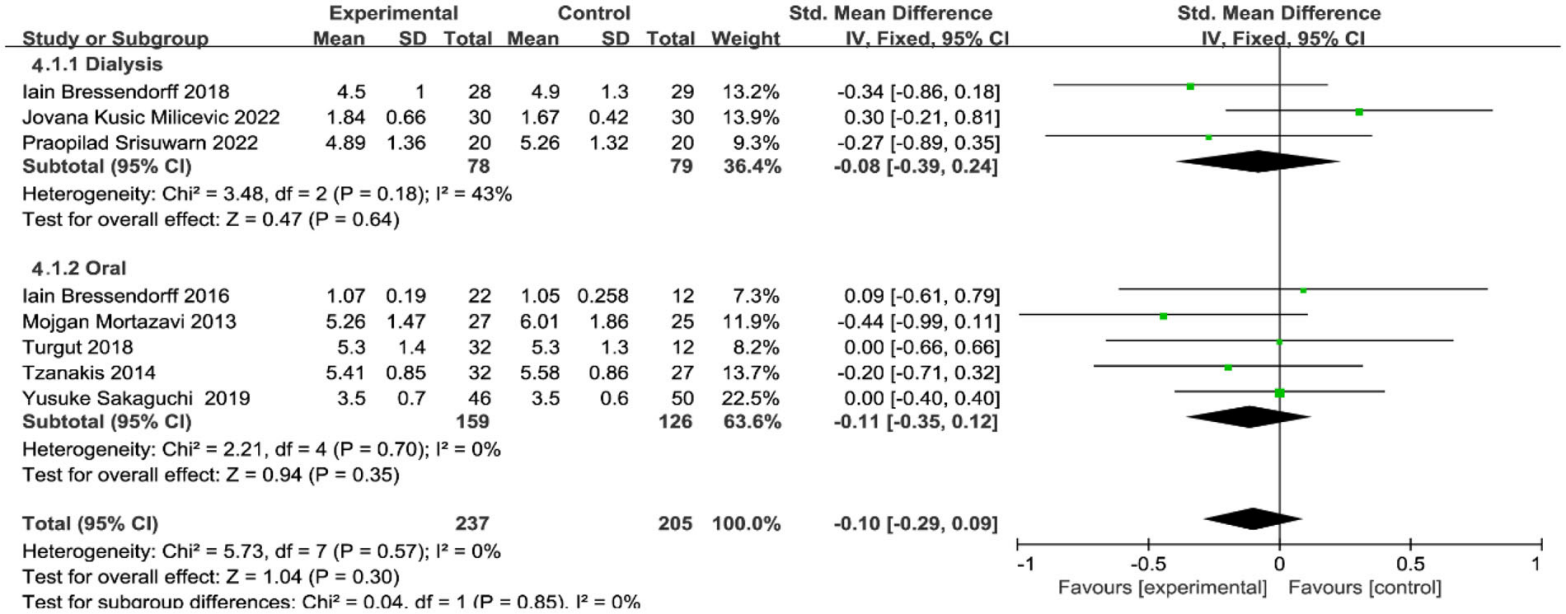
Subgroup analysis of magnesium supplementation on serum P. P, phosphorus.

As shown in Figure 7, eight studies provided measurements of serum P with 237 and 205 patients in the treatment and control groups, respectively. There was no heterogeneity between studies (I2 = 0%, *p* = 0.57), and a fixed-effects model was used. Mg supplementation had no significant effect on serum P levels (SMD = −0.10, 95% CI: −0.29 to 0.09, *p* = 0.30). In further subgroup analysis, Mg supplementation by both medications (SMD=−0.11, 95% CI: −0.35 to 0.12, *p* = 0.35) and dialysis prescription (SMD=−0.08, 95% CI: −0.39 to 0.24, *p* = 0.64) had no significant effect on serum *p* levels.

**Figure 8.**
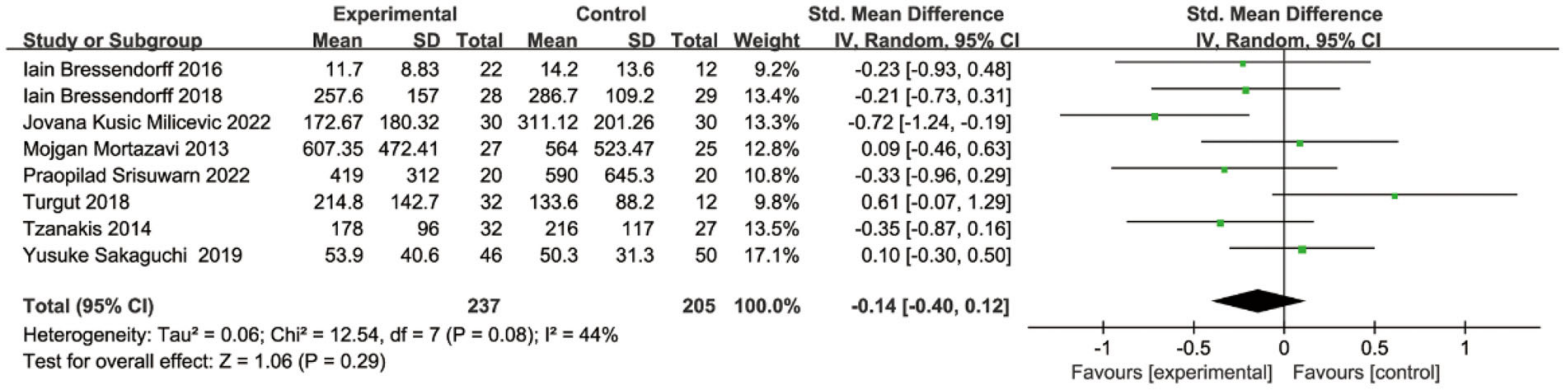
Magnesium supplementation on PTH level. PTH, parathyroid hormone.

Eight studies also provided measurements of PTH, with heterogeneity between studies (I2 = 44%, p = 0.08), using a random-effects model. Mg supplementation had no significant effect on PTH levels (SMD = −0.14, 95% CI: −0.40 to 0.12, *p* = 0.29) (Figure 8). To explore the sources of heterogeneity, sensitivity analysis, and subgroup analysis were performed separately.

Sensitivity analysis was first performed. We removed a study in turn and found no heterogeneity among the remaining studies after removing the Javana et al. study (I2 = 16%, *p* = 0.31). A fixed-effects model was used for analysis, which revealed still no significant effect on PTH levels (SMD=−0.05, 95% CI: −0.28 to 0.17, *p* = 0.66).

**Figure 9.**
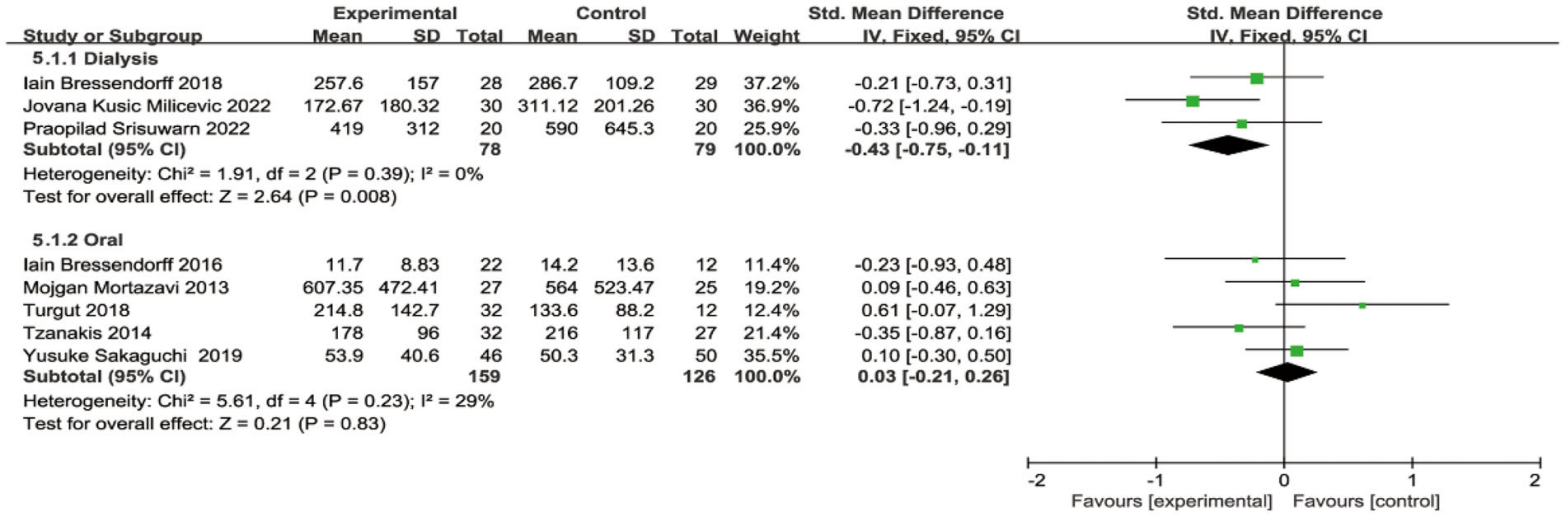
Subgroup analysis of magnesium supplementation on PTH level. PTH, parathyroid hormone.

As shown in Figure 9, subgroup analysis according to the mode of magnesium supplementation, with no heterogeneity in either mode. Oral Mg had no significant effect on PTH levels (SMD = 0.03, 95% CI: −0.21 to 0.26, *p* = 0.83), but increasing the concentration of Mg in the dialysate decreased PTH levels (SMD=−0.43, 95% CI: −0.75 to −0.11, *p* = 0.008) (Figure 9).

### Effect on cIMT

**Figure 10.**
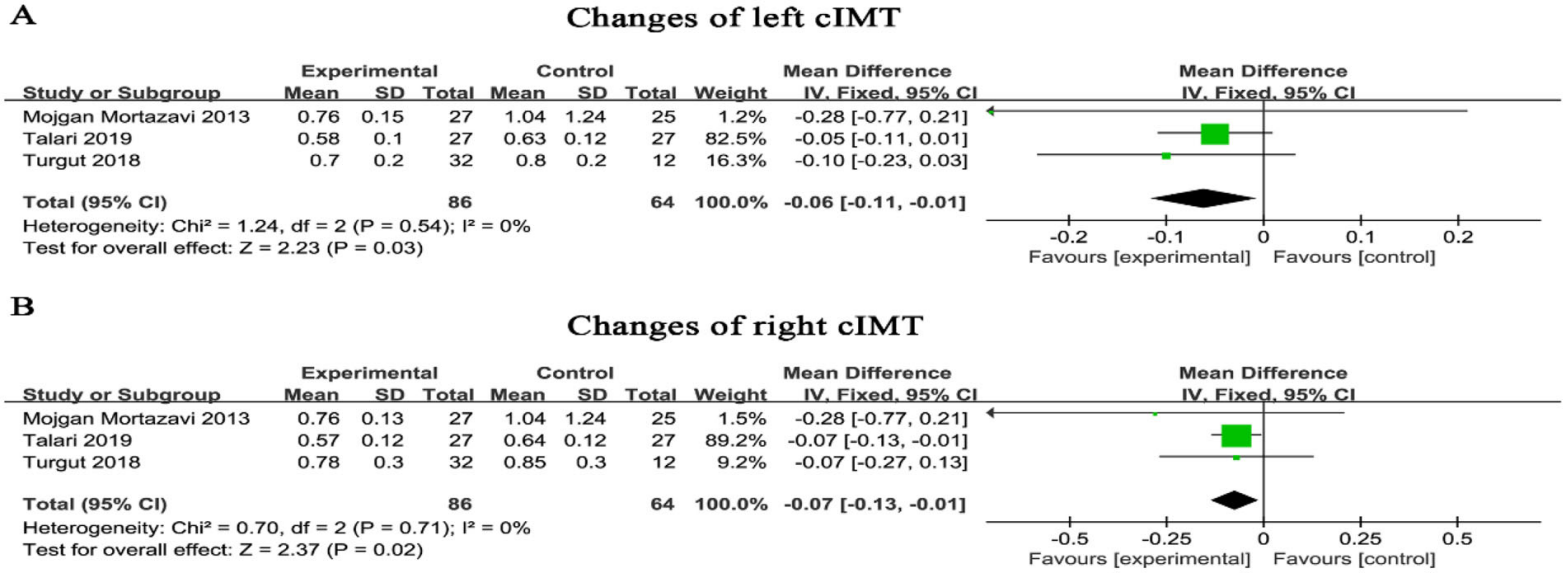
Magnesium supplementation on left (A) and right (B) cIMT. cIMT, carotid intima-media thickness.

Three studies explored the effect of cIMT. cIMT was measured by a carotid artery ultrasound scan. There were 150 patients, including 86 and 64 patients in the trial and control groups, respectively. We explored the effects on the left and right cIMT separately. There was no heterogeneity in either and a fixed effects model was used. The results showed that Mg supplementation reduced left (WMD=−0.06, 95% CI: −0.11 to −0.01, *p* = 0.03) and right (WMD=−0.07, 95% CI: −0.13 to −0.01, *p* = 0.02) cIMT (Figure 10).

### Publication bias and sensitivity analysis

**Figure 11.**
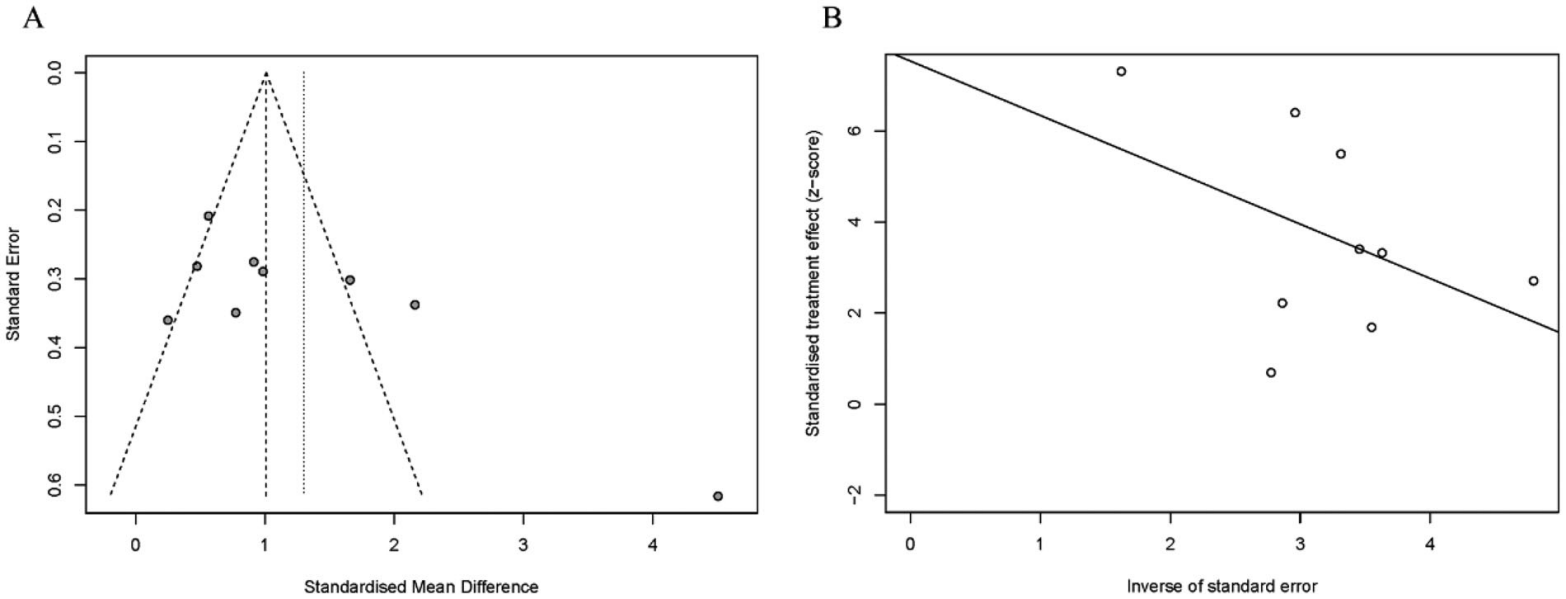
Publication bias assessment. A: Funnel diagram; B: Egger ' s test.

As shown in Figure 11, To evaluate publication bias, we first performed an analysis using a funnel plot, which showed that the funnel plot was not completely symmetrical. We then performed an Egger's test, which showed that there was some publication bias for serum Mg (*p* = 0.038). When removing the literature studies one-by-one, the heterogeneity did not change significantly, indicating that our results are stable.

## Discussion

VC is a common complication of CKD, especially in patients with end-stage renal disease entering dialysis. VC significantly increases the risk of cardiovascular events and all-cause mortality, and is a major cause of mortality in dialysis patients [20]. Early identification, effective treatment and even reversal of VC are of great importance for the long-term survival and improvement of quality of life of patients.

Mg is involved in a variety of physiological functions in the human body. First, we analyzed the effect of Mg supplementation on serum Mg levels. The results showed that Mg supplementation either oral administration or increasing the concentration of Mg in dialysate, especially in dialysate. Low serum Mg levels are associated with increased mortality in patients with CKD. A meta-analysis showed that serum Mg levels were negatively associated with cardiovascular mortality and all-cause mortality in patients with CKD, and that higher Mg levels were associated with lower all-cause mortality and cardiovascular mortality [21]. Animal models suggest that increased dietary Mg inhibits abdominal aortic calcification [22]. Another study including 390 non-diabetic HD patients showed that hypomagnesemia was significantly associated with hand artery VC, serum Mg level is an independent influence on VC in HD patients [23]. Mg supplementation appears to play a positive role in inhibiting VC.

Three of the nine included studies provided quantitative values for the CAC scores, and the other study failed to provide quantitative values for the CAC scores and provided only categorical data; therefore, we analyzed the three studies that provided specific values for the CAC scores. The results showed that Mg supplementation did not slow the progression of VC. A subgroup analysis was performed depending on the form of Mg supplementation and again showed that Mg failed to slow the progression of VC. The results were consistent with a study that had shown no significant effect of high concentrations of Mg dialysate on the progression of VC [15], but again contrary to other studies showing that Mg supplementation slows VC[24]. A systematic review exploring interventions to delay the progression of VC in patients with CKD suggested that Mg supplementation therapy appears to be promising for delaying VC [25]. Our conclusions differ from those of this systematic review. Possible explanations are that on the one hand, although we have included more relevant studies, the overall sample size remains small, and on the other hand, the follow-up period for each study is relatively short. Overall, the effect of Mg supplementation on the treatment of VC is currently uncertain, with inconsistent results between studies. The results of this meta-analysis, which combined all published RCTs studies on the intervention of Mg supplementation on VC, showed no significant effect of Mg supplementation on the progression of VC, but we cannot exclude that this may be limited by the small sample size of the studies conducted so far and the inconsistency of the form of Mg supplementation and the degree of calcification of patients among the studies.

T50 is used to reflect a patient’s propensity for ectopic calcification, and increasing T50 may prevent calcification and reduce the incidence of cardiovascular events [26]. Currently, Mg supplementation has been shown to improve T50 in both small sample studies, but the combined analysis of these two studies in this study found that Mg supplementation did not significantly improve T50. Reviewing the two studies, some heterogeneity can be seen. The heterogeneity may stem from the different forms of Mg supplementation in the two studies, but because only two studies were included, it was not possible to conduct a subgroup analysis to explore the source of heterogeneity exactly at this time.

Patients with CKD have disorders of Ca, P, and PTH metabolism, and it is believed that these metabolic disorders may contribute to the progression of VC [27,28]. Eight studies included in this study provided specific values of Ca, P, and iPTH before and after treatment, so we systematically analyzed whether Mg supplementation affected these indicators. The results showed no significant effect of Mg supplementation on serum P, regardless of whether subgroup analysis was performed.

In a subsequent subgroup analysis, we saw that increasing the Mg concentration in the dialysate reduced the patients’ Ca and PTH levels. A nationally registered cohort study of 142,555 HD patients showed a significant negative association between Mg serum and PTH levels after adjustment for age [8]. The effect of Mg on PTH may be due to the calcium-mimetic effect of Mg stimulating parathyroid calcium-sensitive receptors [29]. In an in vitro study, Mg was shown to decrease the secretion and transcription of PTH in parathyroid cells and to increase the transcription of calcium-sensitive receptors [30]. And Mg causes a decrease in Ca levels in patients probably due to a decrease in PTH levels. Ca and PTH play an important role in the progression of VC. Therefore, this effect of Mg supplementation on Ca and PTH levels may indirectly reflect some possible positive role of Mg in calcification in patients with CKD.

An increase in cIMT is also associated with an increased risk of cardiovascular disease [31]. Clinician usually uses cIMT to evaluate VC. There is a negative correlation between intracellular and serum magnesium status and cIMT [32]. Each of the three studies included in this study showed that Mg supplementation reduced cIMT, and analysis of the three studies combined revealed that Mg supplementation reduced cIMT. Consistent with the results of the previous RCTs that conducted these three. But again, this is contrary to the results of a meta-analysis assessing the effect of Mg supplementation on endothelial function, which showed no significant effect of Mg supplementation on cIMT [33]. However, this meta-analysis was not limited to patients with CKD, it included healthy individuals and patients with hypertension, among others.

There are few RCTs on Mg supplementation to improve VC and its associated markers in patients with CKD, with small sample sizes and inconsistent findings between studies. So, it is very necessary to conduct a comprehensive review to assess the literature related to Mg supplementation for VC in patients with CKD at this stage, to provide a reference for further studies on Mg in patients with VC, or even calciphylaxis. Therefore, this study evaluated the effect of Mg supplementation on VC and related bone metabolic markers in CKD or dialysis patients by means of meta-analysis, but with certain limitations: (1) few clinical trials have been conducted on the effects of Mg supplementation on VC in patients with CKD, with small sample sizes, resulting in a small number of includable literature and limited data. (2) The form of Mg supplementation, dosage, and level of calcification vary between studies, leading to some possible heterogeneity. (3) Although the funnel plot and Egger’s test suggested that the study had some publication bias, the results were shown to be relatively stable by the sensitivity analysis. Moreover, during the study, we tried to reasonably standardize the implementation of the study steps, such as literature search, literature screening, data collection, and analysis, to minimize the generation of bias, and the study results still have some reference value.

## Conclusion

Mg supplementation increased serum Mg levels, and reduced Ca and PTH levels, as well as cIMT, but had no significant effect on CAC scores, a result that may be limited by the small number of relevant interventional studies currently conducted and the small sample size. Therefore, future validation explorations with larger samples and multicenter RCT studies are needed.

## Data Availability

The authors confirm that the data supporting the findings of this study are available within the article.

## Geolocation information

No.190, East Section of Jiannan Road, Youxian District, Mianyang, Sichuan, China
